# Comparison of Acute Responses to Two Different Cycling Sprint Interval Exercise Protocols with Different Recovery Durations

**DOI:** 10.3390/ijerph17031026

**Published:** 2020-02-06

**Authors:** Natalia Danek, Marcin Smolarek, Kamil Michalik, Marek Zatoń

**Affiliations:** 1 Department of Physiology and Biochemistry, Faculty of Physical Education, University School of Physical Education in Wroclaw, 35 Paderewskiego Street, 51-612 Wroclaw, Poland; marcin.cluby@interia.eu (M.S.); marek.zaton@awf.wroc.pl (M.Z.); 2 Departments of Biological and Motor Sport Bases, Faculty of Sport, University School of Physical Education in Wroclaw, 35 Paderewskiego Street, 51-612 Wroclaw, Poland; kamil.michalik@awf.wroc.pl

**Keywords:** sprint interval exercise, cardiorespiratory responses, peak oxygen uptake, blood lactate, perceptual responses

## Abstract

*Background*: Knowledge of acute responses to different sprint interval exercise (SIE) helps to implement new training programs. The aim of this study was to compare the acute physiological, metabolic and perceptual responses to two different SIE cycling protocols with different recovery durations. *Methods*: Twelve healthy, active male participants took part in this study and completed four testing sessions in the laboratory separated by a minimum of 72h. Two SIE protocols were applied in randomized order: SIE_6×10”/4’_—six “all-out” repeated 10-s bouts, interspersed with 4-min recovery; and SIE_SERIES_—two series of three “all-out” repeated 10-s bouts, separated by 30-s recovery and 18-min recovery between series. Protocols were matched for the total work time (1 min) and recovery (20 min). *Results:* In SIE_SERIES_, peak oxygen uptake and peak heart rate were significantly higher (*p* < 0.05), without differences in peak blood lactate concentration and mean rating of perceived exertion compared to SIE_6×10”/4’_. There were no differences in peak power output, peak oxygen uptake and peak heart rate between both series in SIE_SERIES_. *Conclusions*: Two series composed of three 10-s “all-out” bouts in SIE_SERIES_ protocol evoked higher cardiorespiratory responses, which can provide higher stimulus to improve aerobic fitness in regular training.

## 1. Introduction

Interval training can be described as an intermittent exercise, alternating periods of high intensity activity and less intense activity, with the latter facilitating regeneration [1]. One of the most popular is sprint interval training (SIT), which involves generating high levels of power (e.g., on a cycle ergometer) [2]. It usually consists of six “all-out” bouts lasting 10 to 30 s, with the overall time of a training session amounting to 10 to 30 min [3]. Relative to moderate-intensity continuous training (MICT) [3,4], it is considered an effective and time-efficient strategy of improving cardiorespiratory fitness (e.g., maximal oxygen uptake—VO_2_max) and overall physical fitness of people with a sedentary lifestyle. It has, however, not yet been determined what protocol is likely to induce the most sought-after adaptational modifications in regular training.

Buchheit and Laursen [5] defined nine different variables of an interval protocol: work duration, work intensity, work modality, relief duration, relief intensity, series duration, number of series, time between series and between-series recovery intensity, which makes it possible to devise an infinite number of training programs while manipulating the different variables that may well bear a direct influence on cardiorespiratory, metabolic and neuromuscular responses [5]. Previous research based on a two-week training program proved that 10-s bouts (with 4-min recovery) effectively improve cardiorespiratory fitness relative to the “classic SIT” protocol (so called Wingate) composed of 4–6 × 30-s cycle ergometer bouts with 4-min recovery phases [6]. Another research project indicates that reducing the recovery time to one minute leads to an expansion of aerobic and anaerobic efficiency following a 2-week training program similar to the one generated by the 4-min recovery protocol [7]. Furthermore, only the protocol with shorter recovery periods reduced fatigue (power drop) during maximal anaerobic exercise (Wingate test) [7]. In addition, a 3-week training program with 30-s recovery periods brought about a more significant improvement in the endurance test results than training with 80- and 120-s recovery phases [8]. Although long-term chronic adaptation to SIT has been extensively investigated, further research into acute responses to different protocols of individual instances of sprint interval exercise (SIE) appears necessary. Understanding such responses may aid, among others, professional fitness instructors in planning for their interventions.

Responses to exercise are a complex phenomenon encompassing a number of adaptation modifications within the human body organ systems [9]. The contributions of ATP-phosphocreatine (PCr) and glycolysis metabolic pathway are crucial to reaching high exercise intensity, while the oxidative metabolic pathway is essential in maintaining maximum intensity [5] and in PCr resynthesis [10]. Acute responses, e.g., peak power output (PPO), oxygen uptake (VO_2_), heart rate (HR), blood lactate concentration ([La^−^]) and rating of perceived exertion (RPE) in different SIE protocols on a cycle ergometer have been investigated in several research works, however the protocols were matched for total duration of work and recovery by maintaining the established work-to-recovery ratio [11,12]. Some of the research concerned the traditional 4 × 30-s with 4-min recovery protocol [13,14]. Freese et al. [13] reported peak HR 177 ± 12 (beats·min^−1^), peak VO_2_ 38.8 ± 10.6 (mL·kg^−1^·min^−1^) and peak pulmonary ventilation (VEpeak) 107 ± 42 (L·min^−1^), which accounted for around 80% maximal cardiorespiratory functional capacity. In the research of Malta et al. [14], the [La^−^] value obtained of 14.5 ± 1.7 (mmol∙L^−1^) was higher during SIE compared to incremental exercise test (IXT). Moreover, significant correlations have also been established between total work (TW) and maximal oxygen uptake (*r* = 0.51) as well as between TW and maximal aerobic power (MAP) (*r* = 0.89) [14], which indicate that aerobic performance is related withthe ability to repeat short “all-out” efforts. Even though shorter efforts are proven to be more popular with the subjects [15], their response to recovery duration between the 10-s bouts has not been addressed. From the psychological standpoint, understanding those responses is important, since the better perceived (lower RPE) protocols are more frequently picked in regular training.

As stated above, physiological responses can be modulated through interval duration [5]. It has been demonstrated that shorter recovery phases between bouts lead to a stronger response and generate higher VO_2_ and HR values [16]. Other options may also be considered, for instance, splitting the SIE into series. This type of solution is laid out by Hebisz et al. [17], who devised a protocol composed of several series of 30-s “all-out” bouts with 90s recovery time. An active recovery interval of 20 to 30 min between the series allowed for regenerating PPO, HR and [La^−^]. In addition, the protocol enabled the mountain biker subjects to attain higher oxygen uptake and pulmonary ventilation values relative to the incremental exercise test [18]. To our best knowledge, no previous research has been conducted to determine the acute physiological, metabolic and perceptual responses to a protocol consisting of two series of three 10-s “all-out” efforts with short (30 s) recovery periods.

Hence, the present work aims at establishing and comparing the physiological and metabolic responses as well as the perceived exertion of two SIE protocols with 10-s “all-out” bouts but with different recovery durations: SIE_6×10”/4’_, in line with Hazell et al. [6], and SIE_SERIES._ Both protocols were adapted to total work time (1 min) and recovery time (20 min). We assumed that the SIE_SERIES_ protocol composed of two series of 3 × 10-s bouts with a 30-s recovery phase in between and an 18-min interval between the series would trigger higher acute responses. Our second hypothesis was that the duration of the between-series recovery would be sufficient for responses in the second series to be similar to those from the first series. Thirdly, we expected the total work in the two-series protocol, due to greater peak oxygen uptake, would correlate greater with aerobic efficiency indices (VO_2_max and MAP obtained in the incremental exercise test).

## 2. Material and Methods

### 2.1. Participants

Twelve healthy active men participated in this study, who engaged in physical activity for a minimum of 5 h of exercise per week (Table 1). All were physically active, but none of them were participating in competitive sport at a professional level, reporting no cardiovascular and metabolic disease. Throughout the study, physical activity patterns were maintained and no exercise, drink caffeine and alcohol were allowed for 24 h before each test or interval session. The participants were fully informed both verbally and in writing about the study before giving their written informed consent. The study was approved by the local Research Ethics Committee (1/2019) and was performed in line with the Declaration of Helsinki in the Effort Research Laboratory (PN-EN ISO 9001:2001 Certificate).

### 2.2. Study Design

Each participant reported to the laboratory four times, each separated by a minimum of 72h. They were instructed to maintain a sleeping pattern and dietary habits 24 h before all testing sessions in the laboratory. All sessions were conducted by the same researchers and performed in the morning, 2h after breakfast. During the first visit, body mass (kg) and height (cm) were measured using a WPT 200 medical scale (RADWAG, Radom, Poland). An aneroid sphygmomanometer (Riester, Jungingen, Germany) was used to measure blood pressure, followed by an incremental exercise teston the Excalibur Sport cycle ergometer (Lode BV, Groningen, the Netherlands) to determine maximal oxygen uptake (VO_2_max). In the second visit, participants performed a benchmark test (PPO_10_) on a cycle ergometer (Ergomedic Monark 894, Vansbro, Sweden) to measure peak power output (PPO). During the third and fourth visits in the laboratory, the participants performed two SIE sessions (split or not into series) in a randomized order. Physiological (VE, VO_2_, HR) and metabolic ([La^−^]) data were collected before, during and after exercise; perceptual (RPE) data was collected immediately after ending bouts.s

### 2.3. Incremental Exercise Test (IXT)

The incremental exercise test was performed on a stationary Excalibur Sport cycle ergometer (Lode BV, Groningen, the Netherlands) with a linearly increasing load of ~0.28 W·s^−1^ (RAMP protocol) [19] to determine the following parameters: maximal pulmonary ventilation (VEmax), maximal oxygen uptake (VO_2_max), maximal aerobic power (MAP) and maximal heart rate (HRmax). The minimum cadence was 60 rotations per minute (rpm). The test was continued until volitional exhaustion. Recording of respiratory parameters, by a Quark b^2^ device (Cosmed, Milan, Italy), and heart rate (HR) measurements, carried out using an S810 sport-tester (Polar, Kempele, Finland), began three minutes before the exercise and ended five minutes after its completion. VE, VO_2_ and HR were averaged to 30-s intervals and values were calculated per minute. VO_2_max was recorded as the greatest 30-s average at a plateau in VO_2_ values (<1.35 mL·kg^−1^·min^−1^ increase) though the increasing load. The different end criteria used to study the impact on VO_2_max were VO_2_ leveling off, HRmax ≥ 95% of the age-predicted HRmax (220– age) and/or voluntary exhaustion.

### 2.4. Benchmark Test (PPO_10_)

One 10-s “all-out” bout with a load of 7.5% of the participant’s body mass on a cycle ergometer (Ergomedic Monark 894, Vansbro, Sweden) was used to determine peak power output (PPO_10_). The test was preceded by the 10-min warm-up at 60% of the maximal aerobic power obtained in the incremental test, included two 5-s efforts “all-out” in the third and sixth minutes. After warm-up, there was five minutes rest in a sitting position.

### 2.5. Sprint Interval Exercise (SIE) Protocols

**Figure 1:**
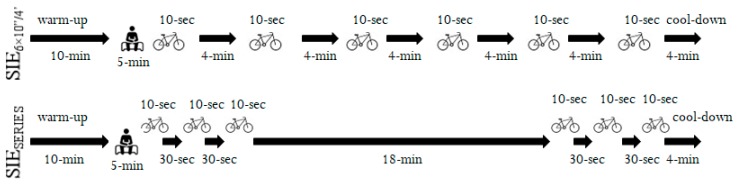
Cycling sprint interval exercise protocols split (SIE_SERIES_) or not (SIE_6×10”/4’_) into series.

During the third and fourth visits in the laboratory, all of the participants randomly performed one of two protocols of sprint interval exercise on the cycle ergometer (Ergomedic Monark 894, Vansbro, Sweden). Both protocols began with a 10-min warm-up (the same as a warm-up before PPO_10_) with a 5-min passive rest after, followed by a 21-min SIE session and a 4-min cool-down (40 min total). Participants performed six 10-s “all-out” bouts, using loads of 7.5% of body mass. Recovery and cooling-down were performed at a cadence of 50 rpm and with a load of 50W. SIE protocols were volume matched for the total duration of work time (1 min) and recovery (20 min) (Figure 1):

(a) SIE_6×10”/4’_—six repeated 10-s “all-out” bouts, separated by 4-min of active recovery [6];

(b) SIE_SERIES_—two series of three repeated 10-s “all-out” bouts, separated by 30-s of active recovery and 18-min of active recovery between series (SERIES I and SERIES II).

To prevent pacing effects, the peak power output produced during the first bout in both protocols was required to equal or exceed 95% of the power during the PPO_10_. The participants were motivated to make the maximum effort in each bout with verbal encouragement.

The obtained results were analyzed for peak power output (PPO), mean power output (MPO) and total work (TW) [20], which were shown as absolute and relative values (per kilogram of body mass). Peak power output and total work were calculated by MCE 2.0 software (MCE, Wroclaw, Poland) for six repetitions in both protocols and separately for three repetitions in the first and second series in SIE_SERIES_. Fatigue index (FI) was determined using the following formula: Fatigue index (FI) = (Peak Power Output – Min Power Output)/Peak Power Output × 100%. Heart rate and respiratory gas analysis (VE, VO_2_) were recorded throughout the SIE session and were averaged to 30-s intervals; values were calculated per minute. VEpeak, VO_2_peak and HRpeak were recorded as the greatest 30-s average.

### 2.6. Blood Lactate Concentrations

Capillary blood was collected from the fingertip immediately before and three min after the incremental exercise test ended, after each repetition in SIE_6×10”/4’_ and three min after SERIES I and SERIES II. The procedure was performed to determine resting and peak blood lactate concentration ([La^−^]) (mmol·L^−1^) with photometric testing (Dr Lange 140 photometer, LP 400 Dr Lange, Berlin, Germany).

### 2.7. Rating of Perceived Exertion

The 15-point Borg scale for the rating of perceived exertion (RPE) was used to measure the level of exertion [21]. The scale starts with “no feeling of exertion” with a rating of 6 and ends with “maximal exertion”, with a rating of 20. The RPE was recorded immediately after each SIE_6×10”/4’_ and SIE_SERIES_ bout. The mean values represented all six bouts in both SIE and separately for the first and second series in the SIE_SERIES_.

### 2.8. Statistical Analysis

The sample size was determined a priori using G*Power 3.1 software (3.1.9.2, Kiel, Germany) [22], the expected effect size was set at (Cohen’s f) 0.85, the α level was set at 0.05, and the power (1-β) was set at 0.8 [23]. The 11 participants in the group were necessary, but to account for potential dropouts, 12 participants were recruited.

Mean oxygen uptake (VO_2_mean) and mean heart rate (HRmean) were calculated for 25 min (1 min of work, 20 min of recovery and a 4-min cool-down) in both SIE protocols. During SIE_SERIES_,VO_2_mean and HRmean were calculated for the two minutes of each series (3 × 10-srepetitions, 2 × 30-s recovery periods + 30s after third bout).

Statistical analysis was performed using Statistica 13.3 software (StatSoft Inc., Tulsa, OK, USA). Data were presented as means ($\bar{x}$) and standard deviations (SD). The distribution of the dataset was screened for normality using the Shapiro–Wilk testand the homogeneity of variances assessed with Levene’s test. The paired Student’s *t*-test was used in the evaluation of the differences in peak and/or mean values of VE, VO_2_, HR, RPE, [La^−^], PPO, MPO, TW and FI between SIE protocols and series in SIE_SERIES_. Comparison of the power output in subsequent bouts was assessed using a one-way repeated measures analysis of variance (ANOVA) in SIE_6×10”/4’_ and two-way (series × number of bouts) ANOVA to determine whether there were any significant differences between both series in SIE_SERIES_. Bonferroni post-hoc tests were performed for pairwise comparisons. Pearson’s linear correlation coefficient were calculated for TW (both SIE protocols) with MAP and VO_2_max. The level of α<0.05 was considered statistically significant. Effect size (ES), that is Cohen’s *d*, was calculated in order to explore practical effect, using the following criteria: 0.1—trivial; 0.2—small; 0.5—medium; 0.8—large [22].

## 3. Results

In testing PPO_10_ peak power output obtained was 11.6 ± 0.9 (W·kg^−1^).

**Figure 2:**
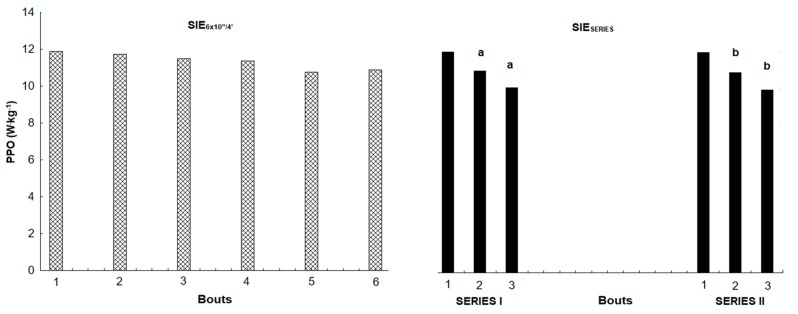
Changes in peak power output for each bout between both sprint interval exercise protocols. ^a^—statistically significant difference between PPO related to first bout in SERIES I (*p* < 0.05), ^b^—statistically significant difference between PPO related to first bout in SERIES II (*p* < 0.05).

Peak power output was not significantly different between SIE_6×10”/4’_ and SIE_SERIES_, and it was similar between SERIES I and SERIES II. There were no statistically significant differences between relative PPO in consecutive bouts during SIE_6×10”/4’_. In SIE_SERIES_, PPO was statistically significantly lower in the second (*p* < 0.05) and third (*p* < 0.001) bouts during the first series and in the second (*p* < 0.001) and third (*p* < 0.001) bouts of the second series (Figure 2), relative to the first bout in the given series. MPO was 3% higher in SIE_6×10”/4’_ (*p* < 0.05, *t* = 2.42, ES = 0.18), a statistically significant increase. Relative MPO was 3% higher in SIE_6×10”/4’_ (*p* < 0.05, *t* = 2.43, ES = 0.36), also a statistically significant increase. Total work was 3.1% higher in SIE_6×10”/4’_ (*p* < 0.05, *t* = 2.42, ES = 0.18), and relative total work was 2.9% higher in SIE_6×10”/4’_ (*p* < 0.05, *t* = 2.31, ES = 0.35). Comparing both series in SIE_SERIES_, MPO was significantly higher (2.8% increase) in SERIES I (*p* < 0.05, *t* = 3.03, ES = 0.11), and relative MPO was significantly higher (2.1%) in SERIES I (*p* < 0.01, *t* =3.11, ES = 0.34). Total work and relative total work between series were significantly higher in SERIES I by 1.3% (*p* < 0.01, *t* = 3.03, ES = 0.08) and by 1.6% (*p* < 0.01, *t* = 3.12, ES = 0.24) (Table 2), respectively.

VO_2_peak was 4.7% higher in SIE_SERIES_ (*p* < 0.05, *t* = 2.55, ES = 0.43). Significant differences were found in VEpeak and HRpeak, being 19.8% (*p* < 0.001, *t* = 7.37, ES = 1.26) and 3.7% (*p* < 0.001, *t* = 4.90, ES = 0.88) higher, respectively, in SIE_SERIES_. There were no differences in resting value, but peak [La^−^] was lower in SIE_SERIES_, without a statistically significant difference (*p* > 0.05). There were no significant differences between the SIE protocols in the mean rating of perceived exertion (*p* > 0.05) (Table 3). As for differences between series in SIE_SERIES_, in the second series HRmean (*p* < 0.001, *t* = 5.21, ES = 0.83) and RPEmean (*p* < 0.03, *t* = 2.57, ES = 0.61) were statistically significantly higher and [La^−^]peak was on the border of statistical significance (*p* = 0.053).

**Figure 3:**
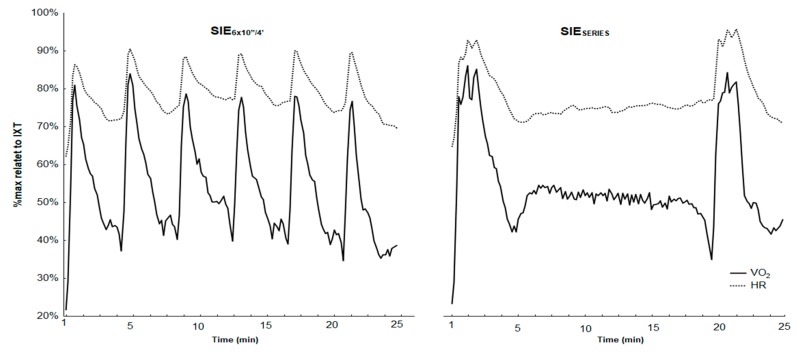
Related values (%max from incremental exercise test) of oxygen uptake and heart rate during and after the SIE_SERIES_ and SIE_6×10”/4’_.

The VO_2_ and HR kinetics during both SIE protocols (regarding to maximal values obtained in the incremental exercise test) were showed in Figure 3.

**Figure 4:**
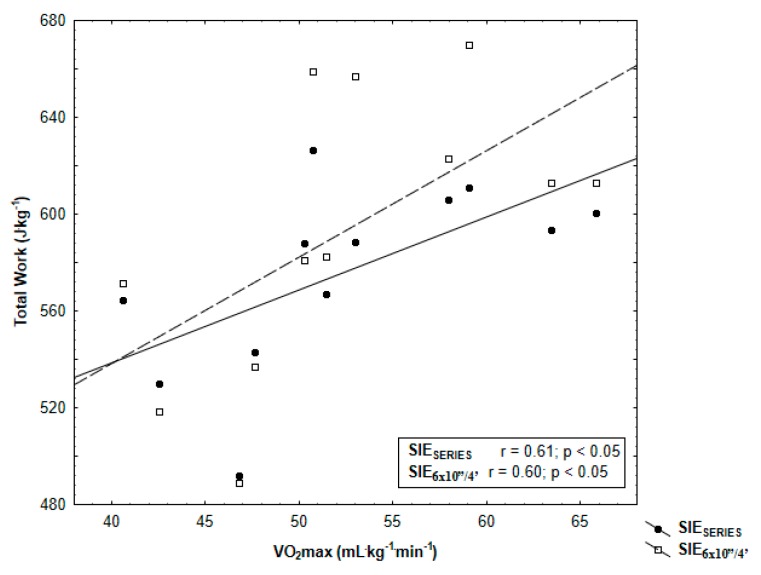
Pearson’s correlation of total work SIE_SERIES_ and SIE_6×10”/4’_ with maximal oxygen uptake (VO_2_max) determined in incremental exercise test.

**Figure 5:**
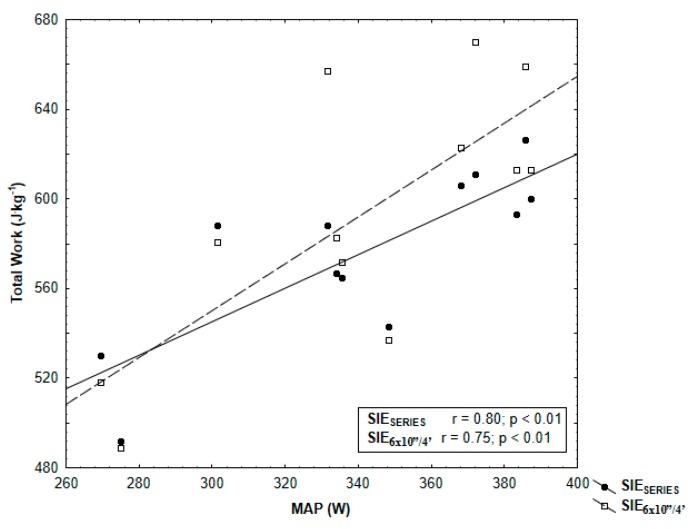
Pearson’s correlation of total work SIE_SERIES_ and SIE_6×10”/4’_ with maximal aerobic power (MAP) determined in incremental exercise test.

Finally, Pearson’s correlation test showed moderate significant (*p* < 0.05) correlations between SIE_SERIES_ and SIE_6×10”/4’_ total work and VO_2_max (*r* = 0.61 and *r* = 0.60, respectively) (Figure 4). SIE_SERIES_ and SIE_6×10”/4’_ total work and MAP were significantly correlated (*r* = 0.80 and *r* = 0.75, *p* < 0.05) (Figure 5).

## 4. Discussion

The optimal SIT protocol has still not been established. Therefore, it is important to find new and alternative protocols that induce the most desirable changes in physical fitness and performance while maintaining positive perception by the exercising people [24]. One of the main findings of this study was a greater cardiorespiratory response during SIE_SERIES_ without differences in peak power, blood lactate concentration and perceptual response in comparison with SIE_6×10”/4’_. Contrary to our second hypothesis, HRpeak and RPEmean were higher and total work performed was lower in the second series of SIE_SERIES_ than in the first series. Additionally, some significant correlations were found between TW with MAP and TW with VO_2_max, which were stronger in SIE_SERIES_.

In both SIE protocols, there were not any differences in peak power output. That indicates a lack of pacing strategy, which was reported in other researchers in sprint interval exercise session [25]. The PPO during SIE_6×10”/4’_ did not decrease, which indicated that a 4-min recovery was sufficient to restore peak power. Previous studies have proven that phosphocreatine resynthesis reaches approximately 90% of the rest value 2 min after an “all-out” bout and allows for the generation of similar power in the next bout [26]. A 30-s recovery between bouts in SIE_SERIES_ reduced power in the second and third efforts in both series, which indicated insufficient PCr resynthesis. The decrease of power in SIE_SERIES_ could explain the increasing concentration of metabolites and peripheral fatigue [27]. It also had an impact on the performance and lower total work (and mean power) in SIE_SERIES_. Reduced PCr availability and continuous attempts to generate peak power in subsequent bouts stimulate both glycolysis and oxidative phosphorylation [28,29].

Greater values of VEpeak, VO_2_peak and HRpeak in SIE_SERIES_ confirmed our first hypothesis. The abovementioned short recovery duration in series may limit PCr resysnthesis; there is presumably greater reliance on aerobic energy in the subsequent bouts [16]. Higher peak VO_2_peak values in SIE_SERIES_ may also result from increased oxygen demand by respiratory muscles, as demonstrated by significantly higher pulmonary ventilation [30], and/or may be caused by the use of oxygen from myoglobin and its replenishment in working muscles [31,32].

Surprisingly, there was no difference in peak lactate concentration between SIE protocols. It has been found that maximal efforts lasting <10 s with shorter recovery time stimulated higher blood lactate concentration [33,34]. This implies that participants had a similar activity of the glycolytic pathway in both sprint interval exercises. According to Gaesser and Poole [35], blood lactate concentration during high-intensity exercise is dependent on relative power output and time. In our research, we obtained lower blood lactate values than Malta et al. [14], who tested 30-s efforts; this is caused by shorter bout times. Another possible explanation is that measurements taken in 3 min after the end of SIE_SERIES_ series were not enough to show the real level of [La^−^] peak, which may require more blood samples, e.g., 5 and 7 min after the completion of the series. These findings showing a lack of differences in both protocols should be explained in future research.

Gibson and Noakes [36] have suggested that performing exercise is dependent on conscious self-regulation or subconscious anticipatory regulation of output power and is determined by cognitive functions and/or motivational factors. In this study, mean ratings of perceived exertion responses to both SIE protocols were similar, despite differences in physiological responses. This is inconsistent with the previous research of Gist et al. [37], who reported that the observed difference in cardiovascular strain can lead to the contrast in perception of effort. On the other hand, lack of differences in blood lactate concentration may also have contributed to the absence of significant differences in mean RPE between SIE protocols, which may be affected by the adjustment of the total work duration (1 min) and recovery (20 min).

According to recommendations of The American College of Sports Medicine (ACSM) regarding physical activity in the development and maintenance of cardiorespiratory, musculoskeletal and neuromotor fitness in healthy people, our peak VO_2_, HR and RPE responses are classified as vigorous (64%–90% of maximal oxygen uptake, 77%–95% maximal HR or rating of perceived exertion as “somewhat hard” or “very hard”, i.e., 14–17) [38]. Thus, the results obtained in our research suggest that the SIE_SERIES_ protocol may be effective in the development of aerobic and anaerobic adaptations similar to SIE_6×10”/4’_, described previously by Hazell et al. [6]. However, similar values of mean oxygen consumption and mean heart rate during 25 min of exercise, but different acute responses, may lead to different long-term training adaptation. It was suggested that the activation of appropriate signaling pathways of mitochondrial biogenesis (e.g., peroxisome proliferator-activated receptor gamma coactivator 1alpha (PGC-1α)) during sprint interval training [39] may depend on the level of power generated during a bout [6], but it may also be caused by glycogenolysis and/or glycolysis [40]. Future studies should investigate the long-term adaptation to SIE_SERIES_ protocol.

In order to adjust two protocols to the duration of bouts and recovery, the authors have applied a constant time of 18-min of active recovery between series. This approach allowed for reproduction of PPO but not total work performed in three bouts. Moreover, there were no differences in peak responses between the both series; however, mean heart rate measured during the 2-min interval in the single series was higher during the second series in SIE_SERIES_, while no differences were found in mean oxygen uptake. These results can be explained by changes in central (cardiac output) [32,41] and peripheral (muscle oxygen extraction) adaptations [32] and/or reduction in plasma volume [42]. These responses undoubtedly influenced participants’ perception of the second series, which seemed to be much harder for them. Thus, our second hypothesis cannot be fully confirmed. According to Tucker [43], decreased work efficiency may be due to the fact that the central nervous system controls the recruitment of motor units depending on the rating of the perceived exertion. Future studies should consider an individual approach to time and intensity of recovery in order to achieve adequate regeneration and the ability to reproduce peak power, total work and level of physiological response in subsequent series with similar RPE. One of the possible approaches is to control the return of pH (H^+^ ion concentration) to the baseline level (>7.30), in accordance with Hebisz et al. [25].

Our results also proved a significant relationship between aerobic performance (maximal oxygen uptake, maximal aerobic power) and total work done during SIE_6×10”/4’_ and SIE_SERIES_. These indicated that participants with higher aerobic performance can perform higher total work during SIE. This can be related to the aforementioned faster PCr resynthesis in comparison with people with lower VO_2_max [10]. Consistent with our third hypothesis, the correlations were higher in the protocol that was split in series. Maximal oxygen uptake is believed to be a physiological index associated with genetic factors [44], whereas maximal aerobic power is a mechanical indicator depending on the level of training [45]. Contrary to the findings of Malta et al. [14], who tested a 4 × 30s protocol with 4-min recovery and a 24-h interval, we observed a stronger correlation between total work done and maximal oxygen uptake measured (*r* = 0.61 vs. *r* = 0.51) in the incremental exercise test and lower correlation between total work done and MAP (*r* = 0.80 vs. *r* = 0.89). The differences reported may be due to the protocols’ application and different participants’ performance levels in both studies.

Although our results are interesting, some limitations need to be addressed concerning the present study. The research group comprised only physically active adult males. Thus, it is difficult to generalize the results obtained for different participants (females, the overweight, the obese, those with sedentary lifestyle, the elderly, etc.). Knowledge of acute responses helps scientists and fitness coaches to design and implement new training methods aimed at achieving the desired physiological changes in physical fitness and health that can be crucial for public health [37]. Future research should include these suggestions in regular training interventions (≥2 weeks) and explain aerobic and/or anaerobic adaptations, hormonal responses and changes in body composition. One such intervention can be an application of only one series (3 × 10s with a 30-s recovery) as an alternative protocol to the recently popular REHIT concept, in which an approximately 10-min training session contains warm-up and cool-down time [24,40] on grounds of time efficiency.

## 5. Conclusions

In summary, the split of six 10-s bouts into series with short recovery periods promotes greater acute cardiorespiratory responses than protocols with constant but longer recovery. Moreover, the participants did not perceive this approach as harder, which may positively induce regular training intervention. These findings have important implications for long-term training adaptation. This seems particularly important in support of the ACSM recommendations that define interval training as the most effective training method [46].

## Figures and Tables

**Table 1:** Participants’ characteristics ($\bar{x}\pm \mathrm{SD}$).

Variables	Values
Age (years)	24.9 ± 4.1
Body height (cm)	181.3 ± 7.7
Body mass (kg)	77.8 ± 10.6
Physical activity (h per week)	7.7 ± 1.6
Systolic blood pressure (mmHg)	123 ± 10
Diastolic blood pressure (mmHg)	70 ± 8
MAP (W)	341.0 ± 41.4
VEmax (L·min^−1^)	148.7 ± 21.1
VO_2_max (mL·kg^−1^·min^−1^)	52.4 ± 7.8
HRmax (b·min^−1^)	193 ± 7
[La^−^] (mmol·L^−1^)	12.8 ± 1.8

MAP—maximal aerobic power; VEmax—maximal pulmonary ventilation; VO_2_max—maximal oxygen uptake; HRmax—maximal heart rate; [La^−^]—blood lactate concentration after incremental exercise test.

**Table 2:** Comparison of mechanical responses between both SIE protocols.

	SIE_6×10”/4’_	SIE_SERIES_	SIE_SERIES_	
			SERIES I	SERIES II
PPO (W)	935.7 ± 161.7	944.5 ± 161.1	936.5 ± 166.0	931.6 ± 149.1
PPO (W·kg^−1^)	12.0 ± 0.9	12.0 ± 0.8	12.1 ± 0.8	12.0 ± 0.9
MPO (W)	771.7 ± 139.3	748.2 ± 123.3 *	754.8 ± 128.3	741.5 ± 118.6 ^#^
MPO (W·kg^−1^)	9.9 ± 1.0	9.6 ± 0.6 *	9.7 ± 0.7	9.5 ± 0.6 ^#^
TW (kJ)	46.3 ± 8.4	44.9 ± 7.4 *	22.6 ± 3.8	22.3 ± 3.6 ^#^
TW (J·kg^−1^)	593.0 ± 57.5	575.9 ± 38.6 *	290.3 ± 19.6	285.6 ± 19.3 ^#^
FI (%)	11.9 ± 8.0	x	15.9 ± 3.9	16.7 ± 5.3

PPO—peak power output; MPO—mean power output; TW—total work; FI—fatigue index; *—statistically significant difference between SIE protocols (*p* < 0.05); ^#^—statistically significant difference between SERIES I and SERIES II in SIE_SERIES_ (*p* < 0.05).

**Table 3:** Comparison of physiological and perceptual responses between both SIE protocols.

	SIE_6×10”/4’_	SIE_SERIES_	SIE_SERIES_	
			SERIES I	SERIES II
VEpeak (L∙min^−1^)	**118.2 ± 18.9**	141.7 ± 18.5 *	137.0 ± 17.7	138.7 ± 19.9
VO_2_peak (mL∙kg^−1^∙min^−1^)	**43.0 ± 4.6**	45.0 ± 4.7 *	44.5 ± 4.5	44.3 ± 3.8
VO_2_mean (mL∙kg^−1^∙min^−1^)	**28.2 ± 2.5**	28.6 ± 2.3	38.7 ± 3.8	39.3 ± 3.5
HRpeak (beats·min^−1^)	**178 ± 8.0**	184 ± 7 *	180 ± 6	184 ± 7
HRmean (beats·min^−1^)	**154 ± 10**	151 ± 11	170 ± 8	177 ± 9 ^#^
[La^−^]peak (mmol·L^−1^)	**13.9 ± 1.8**	13.4 ± 2.3	11.8 ± 1.0	13.3 ± 2.3
RPEmean (6–20)	**15.2 ± 0.9**	15.9 ± 1.4	15.6 ± 2.0	16.6 ± 1.2 ^#^

VEpeak—peak pulmonary ventilation; VO_2_peak—peak oxygen uptake; VO_2_mean—mean oxygen uptake; HRpeak—peak heart rate; HRmean—mean heart rate; [La^−^]peak—peak blood lactate concentration; RPEmean—mean rating of perceived exertion; *—statistically significant difference between protocols (*p* < 0.05); ^#^—statistically significant difference between SERIES I and SERIES II in SIE_SERIES_ (*p* < 0.05).
